# The Problem of the Laurentian Waters

**Published:** 1911-10

**Authors:** 


### The Problem of the Laurentian Waters

At the latest meeting of the Canadian Medical Association, the statement was made, without contradiction, that there was no pure water supply left in Canada. The water problem is one that vitally, as well as economically, affects every community of the globe. Indeed, according to some astronomers whose standing at least entitles them to respectful consideration, it has led, on Mars, to stupendous engineering feats betokening coöperative enterprise superior to anything as yet accomplished on our planet, and resulting in a system of canals and irrigation ditches so that the seasonal stimulation of vegetation is visible across interplanetary space.

If one studies carefully, the maps of the world, one will be struck with the fact that Canada and the north-eastern States are pre-eminently favored with large fresh-water lakes. A couple of lakes well to the north of Russia, Lake Bakal in Siberia, a few large bodies of fresh water in tropical Africa, are the only analogues. Australia, in spite of several large blue blotches on the map, is singularly unfortunate in regard to water supply, owing to the fact that the water sheds are near the coast, resulting in numerous, small rivers tributary to the ocean and a few longer ones which, for the most part, lose themselves in a central desert and vary greatly in volume and potability, according to season.

A water supply derived from large lakes, such as are included in the Laurentian system, is, in many respects, superior to any other, provided, of course, that it is kept free from pollution. Such water is practically unfailing, it varies little in volume or level according to season, it is usually clear, comparatively soft, and free from an excess of mineral matter in solution. From the engineering standpoint and that of commercial use, it is the cheapest and best, largely because a single supply can be lavishly used for all purposes and because, according to standards hardly yet supplanted, sedimentation and filtration, even storage in reservoirs, can be dispensed with.

From the sanitary standpoint, large lakes have the advantage of allowing sedimentation and the death of pathogenic germs from natural extra-corporeal vicissitudes, owing to the slow current. It is, perhaps, worth while to dwell on the fact that we are too prone to consider what artificial factors *kill* germs, and to forget that in unfavorable, natural surroundings, no one of which is positively lethal, germs *die* just as larger plants and animals do. For example, it is altogether likely that if typhoid fever could be prevented for six month, the entire supply of infective bacilli in fomites of various natural kinds, would die out.

Excepting a few local infections practically unknown to European and American physicians, there are only two essentially water-borne infections—typhoid and cholera. With ordinary quarantine precautions at the sea-board, the latter may be considered as practically a dead issue for America. If typhoid could be eliminated—and it will decrease in geometric ratio as sanitary precautions reduce its incidence—even a high grade of pollution of water supplies, would be almost a negligible factor. At the worst, it would produce aggravations of intestinal saprophytosis, and an occasional transmission of entozoa and of infectious germs whose dissemination is mainly by other factors.

The fact has not been sufficiently emphasised that the problem of a pure water supply requires different means of solution according to local conditions. Thus for a small community, it often happens that both purity and water head can best be obtained by guarding the immediate vicinity of one or more springs. For communities without hills and springs, a series of wells, of ordinary depth or Artesian, may be the best solution of the problem. For communities where the only available source is a comparatively small river, uniting the waters of numerous tributaries and ultimately due to surface drainage of an extensive agricultural region,—as in many parts of Europe, New England, and the like,—protection of the water supply against pollution is practically impossible and filtration is the only feasible device. Hamburg, for instance, starting with the water of the River Elbe, which is not much better than that of the old Hamburg Canal of Buffalo, gets a filtrate containing only about 20 bacteria per c.c. and almost absolutely free from pathogens. Yet one can readily imagine that he tastes the chemic pollution of the water.

In the case of a small lake, like Hemlock Lake which supplies Rochester and the lakes, natural and more or less artificial on which New York City draws, it is mainly a question of expense whether to protect the water shed or to filter. When both the community and the lake are small, as in the case of the "Finger Lakes" of New York, the only practical solution of the problem at present, seems to be a fair degree of precaution against human excrement, repeated bacteriologic tests and public warning to boil the water when these are unfavorable or when typhoid is known to exist on the water shed.

In regard to the Laurentian waters, from Lake Superior and Lake Michigan to the Gulf of St. Lawrence, we should not lose sight of the fact that the original water is almost ideal, both from sanitary, hygienic and economic standpoints, nor of the fact that the appreciable danger of infection is of limited radius in lakes. Just what this radius is, has not been definitely determined, and a wide margin of safety should always be allowed. Yet, allowing for the protective force of islands, break-waters and actual currents, the practical impossibility of conveyance of germs through miles of still water, the effects of sedimentation and oxidation, as well as the mere dying out of germs under the natural vicissitudes to which they are subjected, it is probably safe to assume that no conceivable degree of pollution at the head of a lake will endanger the inhabitants at the foot, and that, barring the possible conveyance by boats, water from near the center of a lake is pure.

Probably the most important point is protection of a community against its own and neighboring sewage. Probably, too, allowing for reasonable precaution against off shore currents, lines of traffic, and the like, minor sources of pollution can be ignored and disinfection of sewage will be necessary only for fairly large communities.

Yet it is obvious that the problem differs according to location on the Laurentian waters, and to individual circumstances. For instance a small town or household that can not afford to place an intake far from shore might better employ wells or springs, or some filtration system. Several small communities might unite to protect a strip of shore against local contamination. A large city on an inter-lacustrine river, such as Detroit, Niagara Falls, and the like, would perhaps do well to tap the lake above. Lake Erie, being shallow, frequently becomes turbid so that, even if not grossly contaminated, nor requiring filtration as a protection against typhoid, provision should be made for storage reservoirs of sufficient capacity to tide over periods of roily water. For the long stretch of the St. Lawrence, the problem becomes more difficult, even the height of a town above the water must be considered economically with regard to the availability of inland waters, and, in some instances, a separation of potable and commercial supplies, may be advisable.

The control of water supplies is already recognised, though not without dissent, as a proper subject for governmental interference. The peculiar political constitution of the United States, particularly in regard to the opposing views of state and national sovereignty, as well as the fact that any efficient system of protection of the Laurentian waters must be a subject of international coöperation, complicate the problem. We wish to urge the medical profession to take an active part in the solution of the problem, to appreciate the fact that, all things considered, the region involved has a unique asset of enormous value bestowed upon it by nature, a full understanding of which involves a thorough knowledge of physical geography and of geologic factors largely concerned with the glacial epoch but long antedating it. However necessary it may be for each community to protect itself by prompt action, in accordance with existing conditions, a perfect solution of the problem can be accomplished only by a broad and unselfish conception of the many factors involved. These factors are political, economic and commercial, as well as sanitary and we cannot refrain from expressing the opinion that the ultimate solution of the problem will, to a large extent, involve the use of "raw" water by a population freed from the scourge of typhoid by individual attention to single cases and to fomites of special danger.

